# Adoption of shared decision-making and clinical decision support for reducing cardiovascular disease risk in community health centers

**DOI:** 10.1093/jamiaopen/ooad012

**Published:** 2023-03-10

**Authors:** Jennifer Hauschildt, Kristin Lyon-Scott, Christina R Sheppler, Annie E Larson, Carmit McMullen, David Boston, Patrick J O’Connor, JoAnn M Sperl-Hillen, Rachel Gold

**Affiliations:** OCHIN Inc., Research Department, Portland, Oregon 97228-5426, USA; OCHIN Inc., Research Department, Portland, Oregon 97228-5426, USA; Kaiser Permanente Center for Health Research, Portland, Oregon 97227, USA; OCHIN Inc., Research Department, Portland, Oregon 97228-5426, USA; Kaiser Permanente Center for Health Research, Portland, Oregon 97227, USA; OCHIN Inc., Research Department, Portland, Oregon 97228-5426, USA; HealthPartners Institute, HealthPartners Center for Chronic Care Innovation, Bloomington, Minnesota 55425, USA; HealthPartners Institute, HealthPartners Center for Chronic Care Innovation, Bloomington, Minnesota 55425, USA; OCHIN Inc., Research Department, Portland, Oregon 97228-5426, USA; Kaiser Permanente Center for Health Research, Portland, Oregon 97227, USA

**Keywords:** medical informatics, decision support systems–clinical, decision making–shared, cardiovascular diseases, community health centers

## Abstract

**Objective:**

Electronic health record (EHR)-based shared decision-making (SDM) and clinical decision support (CDS) systems can improve cardiovascular disease (CVD) care quality and risk factor management. Use of the CV Wizard system showed a beneficial effect on high-risk community health center (CHC) patients’ CVD risk within an effectiveness trial, but system adoption was low overall. We assessed which multi-level characteristics were associated with system use.

**Materials and Methods:**

Analyses included 80 195 encounters with 17 931 patients with high CVD risk and/or uncontrolled risk factors at 42 clinics in September 2018–March 2020. Data came from the CV Wizard repository and EHR data, and a survey of 44 clinic providers. Adjusted, mixed-effects multivariate Poisson regression analyses assessed factors associated with system use. We included clinic- and provider-level clustering as random effects to account for nested data.

**Results:**

Likelihood of system use was significantly higher in encounters with patients with higher CVD risk and at longer encounters, and lower when providers were >10 minutes behind schedule, among other factors. Survey participants reported generally high satisfaction with the system but were less likely to use it when there were time constraints or when rooming staff did not print the system output for the provider.

**Discussion:**

CHC providers prioritize using this system for patients with the greatest CVD risk, when time permits, and when rooming staff make the information readily available. CHCs’ financial constraints create substantial challenges to addressing barriers to improved system use, with health equity implications.

**Conclusion:**

Research is needed on improving SDM and CDS adoption in CHCs.

**Trial Registration:**

ClinicalTrials.gov, NCT03001713, https://clinicaltrials.gov/

## BACKGROUND

Use of electronic health record (EHR)-based clinical decision support (CDS) systems and shared decision-making (SDM) systems in primary care settings can improve care quality and management of risk factors for morbidities such as cardiovascular disease (CVD).^1–11^ Care team-facing CDS systems can alert clinic staff when patients have uncontrolled, modifiable CVD risk factors and suggest evidence-based, patient-specific treatment options to address those risks, and SDM systems provide patient-facing summaries of treatment options to enable patient-provider discussions about patient preferences.^12–27^ CV Wizard, the subject of the analyses presented here, is unique in that it provides both CDS and SDM functions: it informs primary care providers *and* patients about treatment options for reducing CVD risk and supports related shared decision-making.

Despite their documented benefits, the use of SDM and CDS systems varies widely.^28–31^ Evidence indicates that adoption of such systems is driven by perceived usefulness, time constraints, how the system fits within established clinic workflows, the frequency with which the use of the system is suggested, and other factors.^29^^,^^32–40^ However, little evidence on SDM or CDS system adoption is available from primary care settings serving socioeconomically vulnerable patients, such as community health centers (CHCs).^4^^,^^13^^,^^26^^,^^41^^,^^42^ In the US, CHCs annually serve 38 million low-income patients, many of whom have complex medical and social needs and uncontrolled CVD risks.^43–47^ Widespread adoption of effective SDM and CDS systems in this setting might incur substantial population health benefits.

The parent study to the analyses presented here tested the effectiveness of CV Wizard, a nonproprietary SDM and CDS system, in reducing CVD risk in the CHC setting.^27^ CV Wizard is a web-based system developed at HealthPartners, a large, nonprofit, integrated care system in Minnesota.^3–7^^,^^48^ At the point of care, the system processes EHR data through evidence-based algorithms that account for a patient’s clinical and demographic characteristics.^49–55^ It then alerts the EHR user to recommend system use for a given patient, that is, those aged 40–75 years with (1) diabetes or CVD and at least 1 uncontrolled CVD risk factor or (2) high reversible CVD risk related to uncontrolled CVD risk factors including (lipids, blood pressure, tobacco, and obesity). CV Wizard’s underlying functionality has been described elsewhere;^3–7^ in brief, the system processes EHR data to assess a given patient’s modifiable CVD risk and determine which modifiable CVD risk factors drive that risk most powerfully. Its output includes the patient’s estimated 10-year CVD risk (likelihood of having a fatal or nonfatal heart attack or stroke in the next 10 years) and the degree to which that CVD risk could be lowered by more effective management of uncontrolled CVD risk factors.

**Figure 1.**
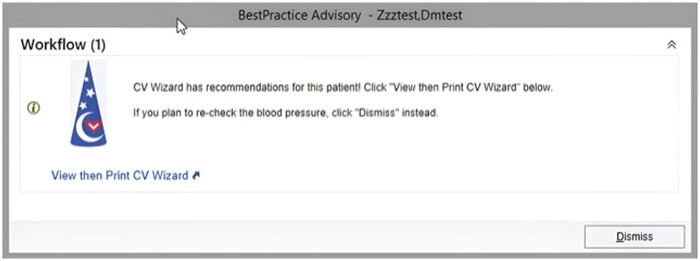
CV Wizard Alert. © 2021 Epic Systems Corporation.

**Figure 2.**
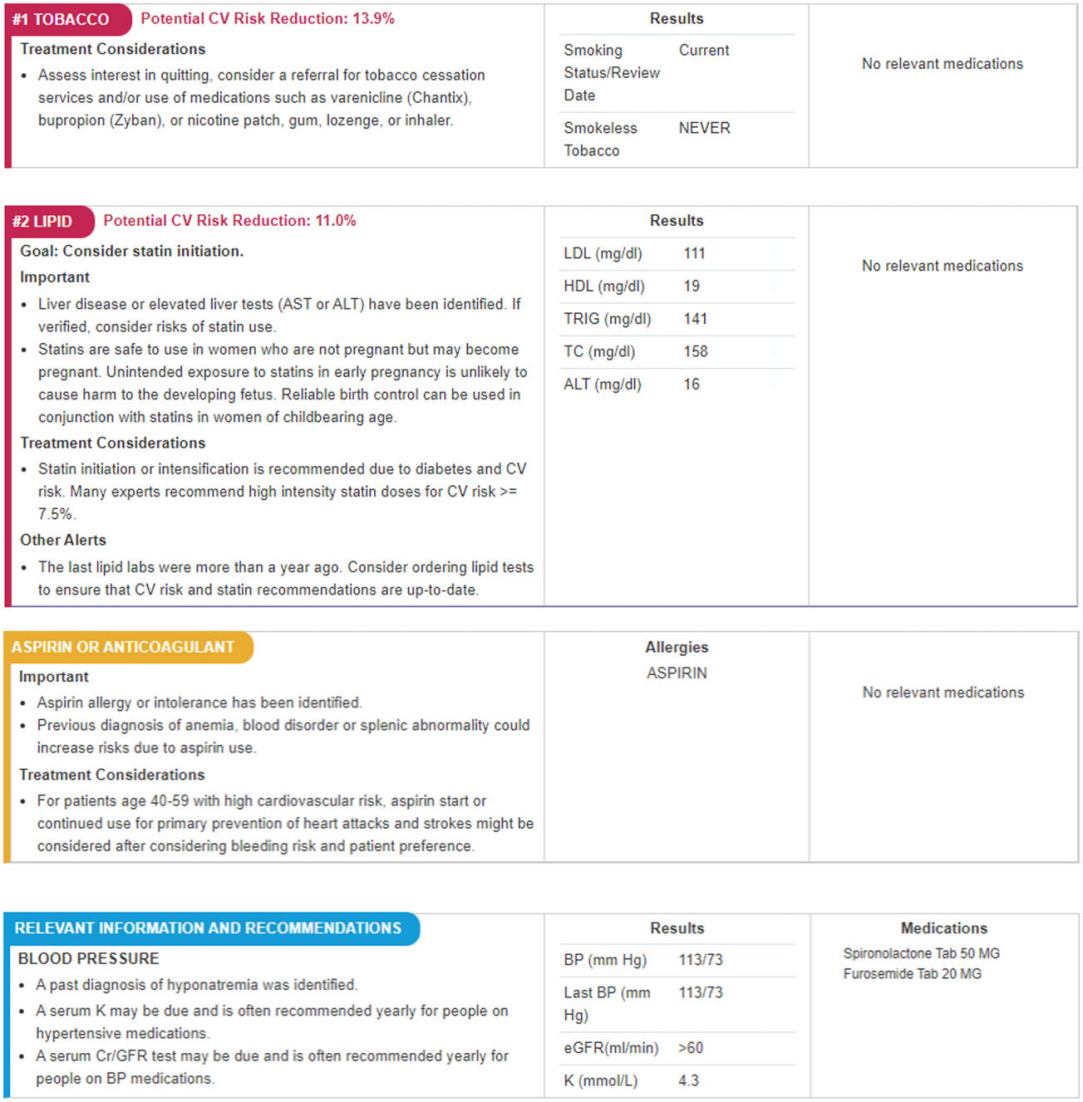
Example of CV Wizard provider view.

**Figure 3.**
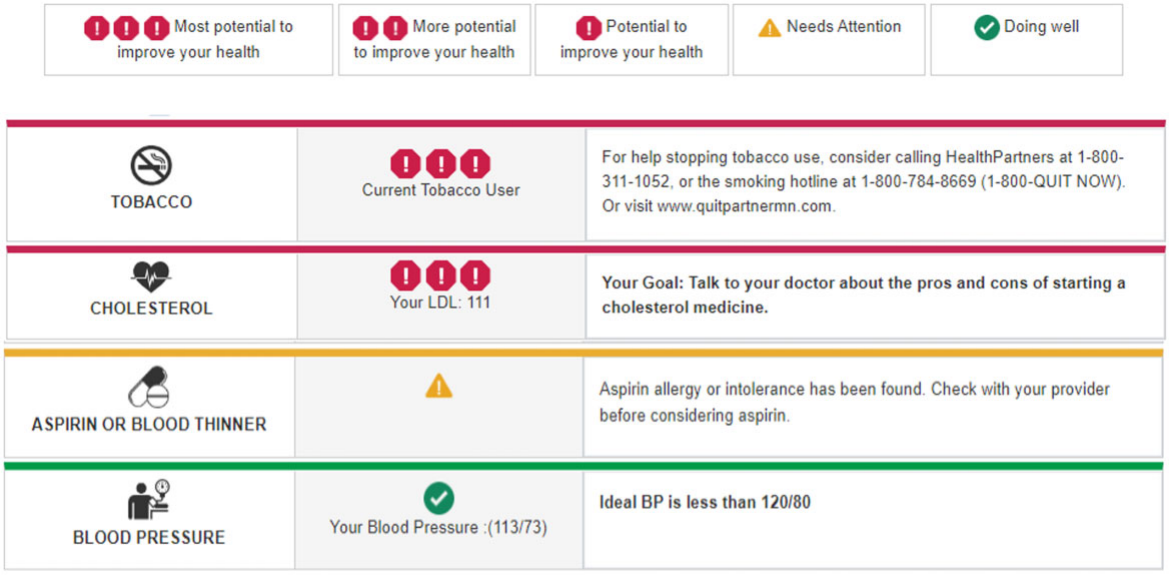
Example of CV Wizard patient view.

During the rooming process, when blood pressure data are entered into the EHR, rooming staff see an alert if system use is recommended for a given patient (Figure 1). Rooming staff can then choose whether to view its output. They can next choose to print out the system’s 1-page, prioritized, individualized risk assessment, which includes output designed for the provider (detailed recommendations) and the patient (in lay language, either English or Spanish) (Figures 2and 3, respectively). Thus, the definition of system use is a care team member viewing and/or printing CV Wizard’s output at an eligible encounter, in response to an EHR alert recommending its use.

Previous studies found that staff in well-resourced integrated care settings printed CV Wizard’s output for 70–80% of target patients,^7^^,^^56^ and that provider satisfaction with the system was high (>85%).^3–7^ Clinic-randomized trials associated its use with improved diabetes management, blood pressure control, and a reduction in reversible CVD risk.^3–7^ Our prior study found a significant reduction in CVD risk among high-risk patients.^27^ Yet in that study, the use of CV Wizard at eligible encounters was far lower than that seen in the integrated care settings where it was first developed and tested.^27^ To understand patterns of CV Wizard adoption in the CHC setting, the analyses presented here assess the associations between patient-, provider-, encounter-, and clinic-level factors and system use at eligible encounters.

## METHODS

### Setting

OCHIN is a nonprofit leader in equitable health care innovation in a growing national provider network.^57^ When this study began in September 2018, OCHIN members consisted of 96 CHC organizations running 493 clinic sites with 761 individual clinics in 14 states. These members utilize a shared OCHIN Epic EHR platform. Table 1 provides the characteristics of the OCHIN clinics included in this analysis.

**Table 1.** Study clinic characteristics

	All clinics (*N* = 42)	All encounters (*N* = 663 407)	Encounters where system fired (*N* = 80 195)		Encounters where system was used (*N* = 21 685)		*P*-value^c^
	*N* (%)	*N* (%)	*N* (%)	System fire rate^b^	*N* (%)	System use rate^b^	
FQHC	30 (71.4)	617 083 (93.0)	78 609 (98.0)	12.7	21 398 (99.0)	27.2	<.001
Rural setting	5 (11.9)	110 798 (16.7)	17 441 (21.8)	15.7	6491 (29.9)	37.2	<.001
Years on current EHR^a^							<.001
0–4	9 (21.4)	42 034 (6.3)	3864 (4.8)	9.2	590 (2.7)	15.3	
5–7	16 (38.1)	183 963 (27.7)	22 450 (28.0)	12.2	7782 (35.9)	34.7	
8	6 (14.3)	109 628 (16.5)	14 714 (18.4)	13.4	3944 (18.2)	26.8	
9–13	11 (26.2)	327 782 (49.4)	39 167 (48.8)	12.0	9369 (43.2)	23.9	
Clinic size by encounters^a^							<.001
100–3000	10 (23.8)	7626 (1.2)	587 (0.7)	7.7	141 (0.7)	24.0	
3001–12 000	11 (26.2)	89 985 (13.6)	9060 (11.3)	10.1	2451 (11.3)	27.1	
12 001–25 000	11 (26.2)	189 311 (28.5)	25 077 (31.3)	13.3	8570 (39.5)	34.2	
25 001–55 000	10 (23.8)	376 485 (56.8)	45 471 (56.7)	12.1	10 523 (48.5)	23.1	
Clinic size by patients^a^							<.001
50–1000	10 (23.8)	7626 (1.2)	587 (0.7)	7.7	141 (0.7)	24.0	
1001–3400	11 (26.2)	97 364 (14.7)	9460 (11.8)	9.7	3254 (15.0)	34.4	
3401–5700	11 (26.2)	185 701 (28.0)	23 128 (28.8)	12.5	7751 (36.7)	33.5	
5701–15 000	10 (23.8)	372 716 (56.2)	47 020 (58.6)	12.6	10 539 (48.6)	22.4	

a Quartiles of encounters and patients seen over entire study period.

b System fire rate is the percent of encounters where system fired out of the total encounters. System use rate is the percent of encounters where system was used out of the fired encounters.

c *P*-value of system ever used versus not used. Chi-squared tests and *t* tests used as appropriate.

### Human subjects review

The Kaiser Permanente Northwest Institutional Review Board (IRB) approved all research activities and monitored study progress. All OCHIN members sign an agreement that their EHR data may be used for research. A Data and Safety Monitoring Board monitored safety outcomes.

### Parent trial

In spring 2018, we recruited 70 OCHIN member clinics run by 15 CHC organizations to the parent trial. We cluster randomized the CHCs 1:1 to intervention or control arms, accounting for CHC size and patient characteristics, as previously described,^27^ and activated CV Wizard in the intervention clinics in September 2018. Six weeks prior, these clinics received a guide on how to use CV Wizard, staff orientation materials, exam room posters, and wizard hats (a playful reminder). Clinic representatives joined several mandatory webinars to review the materials and had the option of attending additional webinars for 6 months after CV Wizard activation to help enhance its adoption. During the comparison period, they received monthly feedback on system-use rates at eligible encounters for their clinic overall and by provider. We offered ongoing consultation on increasing system use about 1 year into the trial.^27^

### Analyses of system use

For the analyses presented here, we calculated the use of the CV Wizard system at eligible encounters in intervention clinics (*n* = 42 clinics, 8 organizations) over an 18-month period (September 20, 2018–March 15, 2020). Eligible encounters included all those at intervention clinics during the study period at which CV Wizard alerted clinic staff that the system had suggestions for that patient. We include only eligible encounters in the analyses reported here but show all encounters and patients seen at study clinics during the study period in Tables 1 and 2.

**Table 2.** Encounter characteristics

	All Encounters (*N* = 663 407)	Encounters where system fired (*N* = 80 195)		Encounters where system was used (*N* = 21 685)		*P*-value^f^
	*N (%)*	*N* (%)	System fire rate^d^	*N* (%)	System use rate^e^	
General characteristics						
Intermediate/high 10-year ASCVD risk (>7.5%)^a^	N/A	55 621 (69.4)	100	16 057 (74.1)	28.9	<.001
Established patient visit	462 695 (69.8)	70 691 (88.2)	15.3	19 787 (91.3)	28.0	<.001
Primary care visit	519 378 (78.3)	69 446 (86.6)	13.4	20 250 (93.4)	29.2	<.001
Provider > 10 min behind schedule^b^	192 018 (28.9)	18 942 (23.6)	9.9	4925 (22.7)	26.0	<.001
Appointment length ≥20 min	522 638 (78.8)	62 838 (78.4)	12.0	18 095 (83.4)	28.8	<.001
System not previously used on patient	N/A	45 173 (56.3)	–	9559 (44.1)	21.2	<.001
Patient demographics						
Female	408 151 (61.5)	44 325 (55.3)	10.9	11 716 (54.0)	26.4	<.001
Age at baseline						<.001
<40	303 407 (45.7)	0	N/A	0 (N/A)	N/A	
40–49	104 762 (15.8)	16 895 (21.1)	16.1	4125 (19.0)	24.4	
50–59	112 431 (17.0)	28 623 (35.7)	25.5	7635 (35.2)	26.7	
≥60	142 807 (21.5)	34 677 (43.2)	24.3	9925 (45.8)	28.6	
Race/ethnicity						<.001
NH-white	245 223 (37.0)	28 416 (35.4)	11.6	8090 (37.3)	28.5	
NH-Black	84 247 (12.7)	15 453 (19.3)	18.3	5929 (27.3)	38.4	
NH-other	45 147 (6.8)	4993 (6.2)	11.1	1239 (5.7)	24.8	
Hispanic	173 334 (26.1)	20 327 (25.4)	11.7	3498 (16.1)	17.2	
Unknown	115 456 (17.4)	11 006 (13.7)	9.5	2929 (13.5)	26.6	
Baseline insurance status						<.001
Medicaid	351 287 (53.0)	30 160 (37.6)	8.6	7233 (33.4)	24.0	
Medicare	107 903 (16.3)	24 483 (30.5)	22.7	7773 (35.9)	31.8	
Other	22 601 (3.4)	4113 (5.1)	18.2	653 (3.0)	15.9	
Private	69 784 (10.5)	7863 (9.8)	11.3	2572 (11.9)	32.7	
Uninsured	111 832 (16.9)	13 576 (16.9)	12.1	3454 (15.9)	25.4	
Baseline FPL category						<.001
≤138% FPL	393 023 (59.2)	49 377 (61.57)	12.6	14 598 (67.3)	29.6	
>138% FPL	144 703 (21.8)	16 779 (20.9)	11.6	4442 (20.5)	26.5	
Unknown	125 681 (18.9)	14 039 (17.5)	11.2	2645 (12.2)	18.8	
Clinic and provider characteristics						
Rural setting	110 798 (16.7)	17 441 (21.8)	15.7	6491 (29.9)	37.2	<.001
Provider type^c^						<.001
Physician	305 958 (46.1)	37 292 (46.5)	12.2	10 619 (49)	28.5	
Advanced practitioner	245 915 (37.1)	29 144 (36.3)	11.9	9197 (42.4)	31.6	
Nurse/MA/other	111 534 (16.8)	13 759 (17.2)	12.3	1869 (8.6)	13.6	

a Categories from ASCVD risk calculator (American College of Cardiology).

b Calculated as the difference in time patient was roomed and the scheduled visit start time.

c Physician category includes MD, DO, ND. Advanced practitioners include PAs, Nurse Practitioners, Midwives. MA: Medical Assistant. Other includes Acupuncturists, BH providers, case managers, Chiropractors, community health workers, lab technicians, and more.

d System fire rate is the percent of encounters where system fired out of the total encounters.

e System use rate is the percent of encounters where system was used out of the fired encounters.

f *P*-value of system ever used vs not used. Chi-squared tests and t-tests used as appropriate.

### Data sources

We obtained data on system use from the CV Wizard web service data repository. Additional EHR-extracted data came from the Accelerating Data Value Across a National Community Health Center Network (ADVANCE) Clinical Research Network, a PCORnet member, including patient, provider, encounter, and clinic characteristics. Researchers interested in accessing the study data can find relevant information at https://ochin.org/research.

### Outcomes

The use of CV Wizard is the main outcome of interest for these analyses, with “use” defined as whether the EHR user chose to view the system output when it alerted the user. As users could opt to just *view* the system output in the EHR, or also *print* it out, these analyses also assessed factors associated with viewing *and* printing the system results (Supplementary File S1, Figure S5). We also report provider survey outcomes.

### Statistical analyses

**Figure 4.**
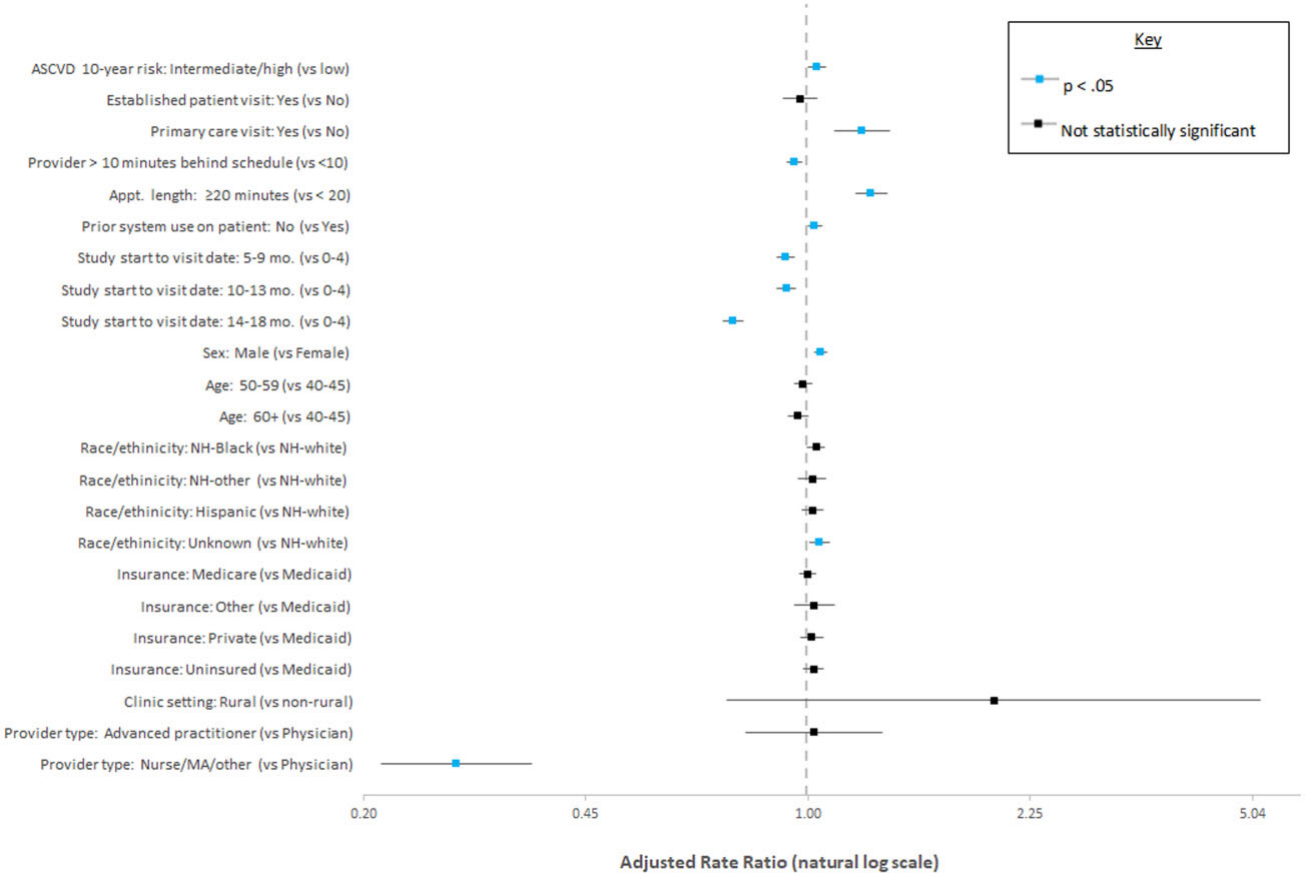
Multivariate regression results. Adjusted rate ratios and 95% confidence intervals of encounter, clinic, provider, and patient characteristics associated with system use (N encounters = 21,685) clustered at the clinic and provider level.

Statistics describe (1) encounters at which CV Wizard alerted users to the patient’s eligibility and (2) the subset of those encounters where the system was used by the clinic (Table 1) and encounter (Table 2). CV Wizard use and nonuse were compared using Chi-squared tests or *t* tests as appropriate. We employed an adjusted, mixed-effects multivariate Poisson regression analysis to identify encounter, clinic, provider, and patient characteristics associated with system use. We included clinic- and provider-level clustering as random effects and controlled for correlations among repeated measurements for individual patients by modeling the variances as an R-sided random effect. We report rate ratios (RR) and 95% confidence intervals for adjusted characteristics in Figure 4. We employed SAS Version 7.15 for all analyses.

### Provider survey

During qualitative data collection for the parent study, we learned that an intervention site clinician had surveyed their providers about CV Wizard. Concurrently, our trial’s plans included direct observations of CV Wizard use during visits to participating clinics. In practice, this was not productive; over 4 days of site visits, we could only observe a few CV Wizard consultations and had limited access to primary care providers for interviews. Looking for another approach to collecting provider perspectives, we decided to modify the clinician-developed survey and administer it to all sites after they had CV Wizard for approximately 1 year. As the survey was not originally planned, we administered in a way designed to limit the survey completion burden on study clinics (details below).

In August 2019, we sent a 9-question provider survey on CV Wizard use (Table 3 and Supplementary File S2) to 9 organizations: the 8 in the trial’s intervention arm and 1 that pilot-tested the system prior to the trial. We programmed the survey in REDCap and sent links to each organization’s study contact, requesting that they forward it to all providers (physicians, nurse practitioners, and physician assistants) in their clinics. Per its introductory text, the survey took no longer than 5 minutes to complete and submission indicated consent to participate. This “low-touch” survey recruitment method utilized a convenience sampling approach that resembled the approach we would have taken had if our originally planned direct observations and informal interviews during clinic visits been more fruitful. Given that we had not initially planned this study activity, we did not want to burden clinics with additional project tasks and therefore did not require survey completion. For these reasons, it was not possible to determine how many providers received the email requesting survey participation. Thematic analysis was performed on the qualitative survey responses (see Supplementary File S3).

**Table 3.** Provider survey descriptive results

Question	Response *N* (%)	
	Yes	No
**Do you know what CV Wizard is?** (*N* = 44)	44 (100)	0 (0)
**Are you using CV Wizard?** (*N* = 44)	32 (73)	12 (27)
**If you are using CV Wizard, what do you like about it?** (*N* = 32)	Checked	Not checked
It is a useful visual aid for patient education	23 (72)	9 (28)
It prioritizes items for CV risk reduction	24 (75)	8 (25)
All CV risk factors are in one place	21 (66)	11 (34)
It reminds me about CV risk issues for the patient	23 (72)	9 (28)
It adds credibility and authority to what I tell patients	19 (59)	13 (41)
Patients like it	3 (9)	29 (91)
Other	1 (3)	31 (97)
**If you are not using it, why not?** (*N* = 12)	Checked	Not checked
It is usually not ready when I go in to see patients	6 (50)	6 (50)
Data is not current	2 (17)	10 (83)
It only pops up for MA, not for me	4 (33)	8 (67)
I don’t get the printouts	3 (25)	9 (75)
My staff is not trained	0 (0)	12 (100)
Not enough time in visits to use it	6 (50)	6 (50)
I don’t find it useful/helpful	4 (33)	8 (67)
I usually ignore pop-ups	2 (17)	10 (83)
I forget about it	6 (50)	6 (50)
Other	2 (17)	10 (83)
**Which of the following would increase your use of CV Wizard?** (*N* = 44)	Checked	Not checked
Having the printout before each CV Wizard-eligible visit	22 (50)	22 (50)
Improving the patient handout	14 (32)	30 (68)
Training on how to use CV Wizard in conversations with patients	8 (18)	36 (82)
Revising the provider view	4 (9)	40 (91)
Making CV Wizard interactive	21 (48)	23 (52)
Revising monthly CV Wizard reports for training/QA monitoring	1 (2)	43 (98)
Me having a better understanding of CV Wizard	3 (7)	41 (93)
MAs having a better understanding of CV Wizard	10 (23)	34 (77)
Revised workflows	8 (18)	36 (82)
Other	10 (23)	34 (77)

## RESULTS

A total of 80 195 encounters at the parent study’s 42 intervention clinics were eligible for use of CV Wizard; clinics used CV Wizard in 27% of these encounters (Table 1).

Over 70% of study clinics were federally qualified health centers (FQHCs), and 98% of eligible encounters occurred in these clinics (Table 1). Twelve percent of study clinics were in a rural area and 22% of eligible encounters were in these clinics.

Almost 70% of the eligible encounters were with patients who had intermediate or high 10-year CVD risk (Table 2). Most were primary care (87%) and established patient (88%) visits. Thirty-six percent of eligible encounters were with patients aged 50–59 years, 43% with patients aged 60–74 years, and 55% with female patients; 35% were with patients who were non-Hispanic white, 25% Hispanic, and 19% non-Hispanic Black. Thirty-eight percent of eligible encounters were with patients whose insurance status at baseline was Medicaid, and 31% were with those covered by Medicare. Most eligible encounters (62%) were with patients from households at ≤138% of the federal poverty level. Providers were running >10 minutes behind schedule at 24% of eligible encounters, and 78% of eligible encounters were scheduled for ≥20 minutes. Almost half (46%) of these encounters were with a physician, and 36% were with an advanced practitioner (Table 2).

Figure 4 presents factors associated with the increased likelihood of CV Wizard use (viewing only *or* viewing and printing system output). Likelihood of system use was significantly higher: at encounters for patients with intermediate to high 10-year ASCVD risk compared to those with lower risk (RR 1.04, 95% CI 1.01–1.07, *p *=* *.02); at primary care visits (RR 1.22, 95% CI 1.11–1.35, *p *<* *.001); at encounters scheduled for ≥20 minutes (RR 1.26, 95% CI 1.19–1.34, *p *<* *.001); with male patients (RR 1.05, 95% CI 1.03–1.07, *p *<* *.001); and with patients for whom there was no previous use of the system (RR 1.03, 95% CI 1.00–1.05, *p* = .03). Likelihood of system use was significantly lower at encounters where providers were >10 minutes behind schedule (RR 0.96, 95% CI 0.93–0.98, *p *<* *.01), and those occurring 5–13 months (RR 0.93, 95% CI 0.90–0.96, *p *<* *.001) and 14–18 months (RR 0.76, 95% CI 0.74–0.79, *p *<* *.001) after training on system use was provided. Likelihood of system use was also significantly lower at encounters with a nurse or medical assistant compared to those with a physician (RR 0.28, 95% CI 0.21–0.37, *p* < .001).

Most factors associated with viewing *and* printing CV Wizard's output were similar (Supplementary File S1). The system’s results were significantly more likely to be viewed *and* printed at encounters in rural clinics compared to urban clinics (RR 3.97, 95% CI 1.10–14.29, *p *=* *.04), and less likely in those with patients aged 60 years or older compared to younger patients (RR 0.96, 95% CI 0.92–0.99, *p *=* *.03).

### Provider perspectives on system use and nonuse

In total, 44 providers submitted a survey response. At least 1 provider from 6 of the 9 study organizations responded (range = 1–22 provider responses per organization).

All surveyed providers reported knowing what CV Wizard was, and 73% (32/44) reported using the system. Of those, most liked that the system prioritized factors for CV risk reduction (24/32, 75%), provided a useful visual aid for patient education (23/32, 72%), reminded the provider about patient-specific CVD risks (23/32, 72%), consolidated data on all major uncontrolled CVD risk factors in 1 place (21/32, 66%), and added credibility to what the provider tells the patient (19/52, 59%). Commonly noted reasons for nonuse of the system output included not enough time during the visit (6/12, 50%), the output not being ready when the provider saw the patient (6/12, 50%), and forgetting to use it (6/12, 50%). Two nonusers reported that they got CVD risk estimates from other sources (eg, a different risk calculator); see Table 3 and Supplementary File S2.

## DISCUSSION

This is one of the first assessments of the adoption of CV Wizard, which targets CVD risk management, in the CHC setting. Most prior research on patterns of SDM or CDS adoption comes from well-resourced care settings. The findings presented here contribute novel evidence on such adoption in CHCs, which serve socioeconomically vulnerable populations and operate with very limited resources. Given the potential health benefits of SDM and CDS use—that is, our previous study showed a significant reduction in 10-year CVD risk among CHC patients with high CV risk when the CV Wizard system was used^27^—increasing their use in CHCs might reduce health disparities.

While elements of these findings align with those from better-resourced care settings, their implications are different in CHCs. Here, system use was lower when the provider had less time, as is consistent with prior research.^11^^,^^58–60^ CHC patients are generally more medically and socially complex than those in other care settings, so may have competing clinical demands more often than those in other settings, making it more likely that CVD risk management cannot be prioritized at a given encounter. System use was lower in shorter encounters; <20 minutes may not be enough time to address a patient with complex clinical needs, let alone discuss CVD prevention. Yet, CHCs may face especially difficult barriers to supporting longer encounters. State and federal policies determine their rates of reimbursement for provided care, which are not commensurate with payment for similar care covered by private insurance;^61^ they are thus incentivized to have more—and shorter—encounters per day. Adjustments to state and national policies about how CHCs are reimbursed for the care they provide—including changes that would allow for longer visits—might result in increased CDS and SDM adoption. Given the potential impact that the use of such systems can have on the provision of guideline-concordant care, payment structures that support their use might ultimately be cost-saving and improve health equity.

Similar to research from better-resourced settings, we identified barriers related to fitting system use into existing workflows.^11^^,^^58^^,^^59^^,^^62^ In survey results, providers often noted that system use did not occur if rooming staff did not print its output at the start of the encounter, which limited provider-patient discussions about CVD risk management options. The CV Wizard system is designed to be used by rooming staff printing its results, as the printed output enables point-of-care provider-patient discussions about CVD risk management options. If CHCs are unable to afford printers in each exam room, tool use workflows will involve more steps and rooming staff might be less inclined to print results. If so, this barrier might also be improved by ensuring that CHCs receive adequate funding to pay for and maintain printers.

These results have novel implications about how such systems are designed in terms of which patients they target. Here, the use of CV Wizard was more likely at encounters with patients who had a higher baseline 10-year CVD risk, or who were older and/or male. Providers may have suspected that these patients had high CVD risk even before reviewing system output, and thus been more inclined to use the system in these cases. This suggests that the use of CV Wizard might increase by limiting the alert-suggesting tool use to higher-risk patients. Similarly, the system was less likely to be used in encounters with clinic staff other than physicians or advanced practitioners, perhaps because such encounters were for specific types of care and therefore not conducive to discussing CVD risk. Additionally, since the recommendations often involve medication initiation or changes, prescribing providers would be more apt to use it. Focusing the system on a smaller proportion of patients, or only at encounters led by a prescribing provider, might thus increase its perceived usefulness and fit with clinical priorities and thus its rates of adoption. Research is needed to test this hypothesis. Another approach might be to enhance the automation of future CDS tools, for example, by programming them to automatically order medications to save provider time. However, this approach might circumvent the system’s SDM function, which might undermine its overall utility.

Another novel implication is that more effective and frequent training might have enhanced system use. Clinical staff reported that they forgot about the system, did not remember how to use it, or would have preferred more training in its use. Analyses showed that staff used the system less often as time passed after initial training. This result might reflect the high staff turnover experienced in CHCs, as new staff were not necessarily oriented to the system. Furthermore, this CDS and SDM system involves steps taken by both the rooming staff and clinician (ie, rooming staff print the tool’s output to support the provider discussing it with the patient). This has implications about how to conduct staff training in the use of CDS and SDM systems. Refresher trainings and orientation of onboarding staff may be necessary to enhance system adoption. Coordinated training specific to different clinic roles may also be helpful. In addition, better training might make the system easier to use when providers are time-constrained, per prior discussion. Future research should focus on how to optimize trainings in CDS and SDM system use in CHCs, although substantial barriers would still exist regarding ongoing trainings. Most critically, CHCs do not generate income when their staff spend time in trainings. Reimbursement policies must be redesigned to incentivize CHC participation in training to overcome this barrier.

### Limitations

There are substantial limitations to the survey methods that are important in interpreting results. As noted previously, we developed and administered the survey in response to information obtained during qualitative data collection for the parent study. We did not intend for it to be a primary data source; rather, the goal was to provide ad hoc insight regarding system use patterns. We did not require survey participation, and research staff did not know (1) which sites forwarded the invitation to their providers or (2) how many providers were invited to participate at the sites that did share it. Therefore, we were unable to determine a response rate. Further, we did not survey rooming staff, whose role in CV Wizard use was key. In addition, about half of the survey results came from a single organization. For these reasons, the survey findings should be considered exploratory.

## CONCLUSIONS

Achieving SDM and CDS’ potential impact on health outcomes will require understanding and addressing myriad barriers to such systems’ systematic adoption. This is likely to be especially challenging in safety net CHCs, where patient complexity is high and reimbursement rates are not commensurate with those in better-resourced settings. Future research should explore the most critical barriers to SDM and CDS adoption in the CHC setting and the most effective strategies for addressing these barriers. Until such knowledge is generated, the adoption of technologies like SDM and CDS systems likely will occur primarily in well-resourced care settings, with the potential to exacerbate health disparities.

## Supplementary Material

ooad012_Supplementary_Data

## Data Availability

Raw data underlying this article were generated from multiple health systems across institutions in the ADVANCE Network; restrictions apply to the availability and re-release of data under organizational agreements.
