# Supplementary material for: Adoption of shared decision-making and clinical decision support for reducing cardiovascular disease risk in community health centers
Source: JAMIA Open. 2023 Mar 10;6(1):ooad012. doi: 10.1093/jamiaopen/ooad012 (PMC10005607; doi:10.1093/jamiaopen/ooad012)
Supplement: ooad012_Supplementary_Data [file ooad012_supplementary_data.zip › CVW_ProviderSurvey.pdf]

CV WIZARD PROVIDER SURVEY

1. **Are you a physician, nurse practitioner or physician assistant who provides care to adult patients?**
   □Yes □No

   If Q1 Answer is NO:

   END SURVEY and display text: "At this time, we are only requesting feedback from clinicians providing adult care. Thank you for your willingness to complete the survey."

   If Q1 Answer is YES:

   PROCEED with survey.

2. **Do you know what CV Wizard is?**
   □Yes □No

3. **Are you using CV Wizard?**
   □Yes/Sometimes □No

   If Q3 Answer is Yes/Sometimes:

   4. **If you are using CV Wizard, what do you like about it? [check all that apply]**
      □ It is a useful visual aid for patient education
      □ It prioritizes items for CV risk reduction
      □ All CV risk factors are in one place
      □ It reminds me about CV risk issues for the patient
      □ It adds credibility and authority to what I tell patients
      □ Patients like it
      □ Other____________________

   If Q3 Answer is NO:

   5. **If you are not using it, why not? [check all that apply]**
      □ It is usually not ready when I go in to see patients
      □ Data is not current
      □ It only pops up for MA, not for me
      □ I don't get the printouts
      □ My staff is not trained
      □ Not enough time in visits to use it
      □ I don't find it useful/helpful
      □ I usually ignore pop-ups
      □ I forget about it
      □ Other____________________

6. **Which of the following would increase your use of CV Wizard [check all that apply]**
□ Having the printout before each CV Wizard-eligible visit
□ Improving the patient handout
□ Training on how to use CV Wizard in conversations with patients
□ Revising the provider view
□ Making CV Wizard interactive (to show risk change with risk factor changes, e.g., quitting smoking)
□ Revising monthly CV Wizard reports to assist with training and quality monitoring
□ Me having a better understanding of CV Wizard
□ MAs having a better understanding of CV Wizard
□ Revised workflows
□ Other ___________________________________

7. **When CV Wizard fires, how often does it influence what you discuss with the patient?**
□ All of the time
□ Most of the time
□ Sometimes
□ Rarely
□ Never

8. **Please share any other comments or suggestions you may have:**

[open text box]

9. **Please indicate at which clinic you mainly practice.**

□ **(Clinic name 1)**
□ **(Clinic name 2)**
□ **(Clinic name 3)**
□ **(Clinic name 4)**
□ **(Clinic name 5)**

[**Submit Survey** button]
