# Supplementary figures and images for: Adoption of shared decision-making and clinical decision support for reducing cardiovascular disease risk in community health centers
Source: JAMIA Open. 2023 Mar 10;6(1):ooad012. doi: 10.1093/jamiaopen/ooad012 (PMC10005607; doi:10.1093/jamiaopen/ooad012)

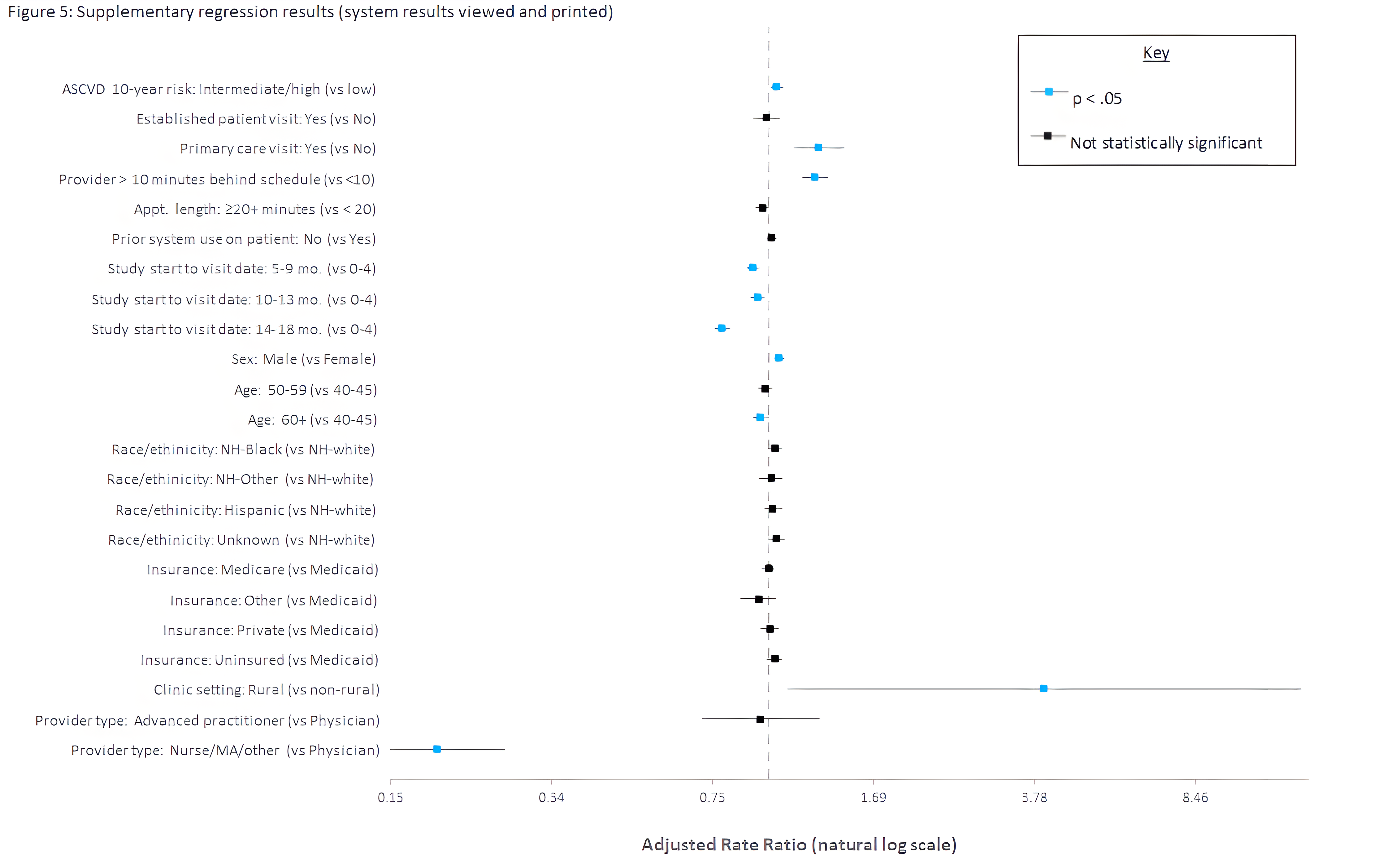

Supplement: ooad012_Supplementary_Data [file ooad012_supplementary_data.zip › fig 5_supplementary file.tif]

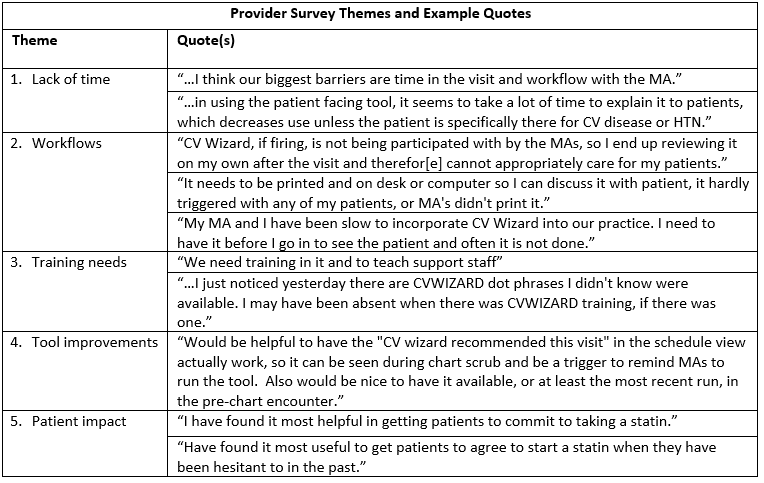

Supplement: ooad012_Supplementary_Data [file ooad012_supplementary_data.zip › Provider Survey Themes and Example Quotes.PNG]
